# Handbook of Materia Medica, Pharmacy and Therapeutics

**Published:** 1887-07

**Authors:** 


**Handbook of Materia Medica, Pharmacy and Therapeutics,** including the Physiological Action of Drugs, the special Therapeutics of Disease, official and extemporaneous Pharmacy and minute Directions for Prescription Writing. By SAMUEL O. L. POTTER, A.M., M.D., Professor of the Theory and Practice of Medicine in the Cooper Medical College of San Francisco; author of "Quiz Compends of Anatomy and Materia Medica," an index of comparative therapeutics and a study of speech and its defects; late A. A. Surgeon U. S. Army. Philadelphia: P. Blakiston, Son & Co., 1012 Walnut street. 1887.

This work, as seen by its title, embraces much that is new to the profession, including, as it does, well-written and concise articles on extemporaneous pharmacy, as well as official. The parts on physiological action of medicines is well-written and comprehensive. The portion of the book relating to special therapeutics of disease is especially valuable, as it is so concise and to the point. Taking it all in all, this is a book well worth having. Hence, we hope it will find a high place among the recent manuals of its class, as it has many merits and few, if any, demerits.

